# The density of mast cells c-Kit^+^ and tryptase^+^ correlates with each other and with angiogenesis in pancreatic cancer patients

**DOI:** 10.18632/oncotarget.19716

**Published:** 2017-07-31

**Authors:** Michele Ammendola, Cosmo Damiano Gadaleta, Adam Enver Frampton, Tullio Piardi, Riccardo Memeo, Valeria Zuccalà, Maria Luposella, Rosa Patruno, Nicola Zizzo, Pietro Gadaleta, Patrick Pessaux, Rosario Sacco, Giuseppe Sammarco, Girolamo Ranieri

**Affiliations:** ^1^ Department of Medical and Surgical Sciences, Clinical Surgery Unit, University of Catanzaro “Magna Graecia” Medical School, Viale Europa-Germaneto, Catanzaro, Italy; ^2^ HPB Surgical Unit, Department of Surgery and Cancer, Imperial College, Hammersmith Hospital, London, UK; ^3^ Department of General, Digestive and Endocrine Surgery, Hopital Robert Debre, Centre Hospitalier Universitaire de Reims, Universite de Reims Champagne-Ardenne, Reims, France; ^4^ Pathology Unit, “Pugliese-Ciaccio” Hospital, Catanzaro, Italy; ^5^ Cardiovascular Disease Unit, “San Giovanni di Dio” Hospital, Crotone, Italy; ^6^ Chair of Pathology, Veterinary Medical School, University “Aldo Moro”, Bari, Italy; ^7^ Interventional Radiology Unit with Integrated Section of Traslational Medical Oncology, National Cancer Research Centre, “Giovanni Paolo II”, Bari, Italy; ^8^ Hepato-Biliary and Pancreatic Surgical Unit, General, Digestive and Endocrine Surgery, IRCAD, IHU Mix-Surg, Institute for Minimally Invasive Image-Guided Surgery, University of Strasbourg, 1 place de l'Hôpital, Strasbourg, France

**Keywords:** angiogenesis, mast cells, tryptase, c-Kit-receptor, pancreatic ductal adenocarcinoma

## Abstract

Literature data suggest that inflammatory cells such as mast cells (MCs) are involved in angiogenesis. MCs can stimulate angiogenesis by releasing of well identified pro-angiogenic cytokines stored in their cytoplasm. In particular, MCs can release tryptase, a potent *in vivo* and *in vitro* pro-angiogenic factor. Nevertheless, few data are available concerning the role of MCs positive to tryptase in primary pancreatic cancer angiogenesis. This study analyzed the correlation between mast cells positive to c-Kit receptor (c-Kit^+^ MCs), the density of MCs expressing tryptase (MCD-T) and microvascular density (MVD) in primary tumor tissue from patients affected by pancreatic ductal adenocarcinoma (PDAC). A series of 35 PDAC patients with stage T_2-3_N_0-1_M_0_ (by AJCC for Pancreas Cancer Staging 7^th^ Edition) were selected and then undergone to surgery. Tumor tissue samples were evaluated by mean of immunohistochemistry and image analysis methods in terms of number of c-Kit^+^ MCs, MCD-T and MVD. The above parameters were related each other and with the most important main clinico-pathological features. A significant correlation between c-Kit^+^ MCs, MCD-T and MVD groups each other was found by Pearson t-test analysis (r ranged from 0.75 to 0.87; p-value ranged from 0.01 to 0.04). No other significant correlation was found. Our *in vivo* preliminary data, suggest that tumor microenvironmental MCs evaluated in terms of c-Kit^+^ MCs and MCD-T may play a role in PDAC angiogenesis and they could be further evaluated as a novel tumor biomarker and as a target of anti-angiogenic therapy.

## INTRODUCTION

Mast cells (MCs), following c-Kit activation, stimulates angiogenesis in various types of human tumors. Prot-oncogene c-Kit mutation is identified in animal and human tumors including dog mastocytoma, gastrointestinal stromal tumor (GIST) and pancreatic cancer [1–4].

In 1986 Besmer P et al found the viral *v-kit* sequence and it was demonstrated that this gene was involved in the pathogenesis of the feline sarcoma virus. One year later the cellular corresponding homologue c-Kit gene was also discovered and it was demonstrated that the protein codified by the above gene was a membrane tyrosine kinase receptor [5].

The ligand for c-Kit receptor (c-Kit-R) is the Stem Cell Factor (SCF) and Kit-R is expressed by several cellular kind such as hematopoietic precursor, germ cells, interstitial cells of Cajal, melanocytes, and mainly MCs. With special reference to MCs the activation of c-Kit-R driven the main important cellular function and in particular: survival, proliferation and differentiation [6].

It is now well established that MCs contains a lot of stimulating angiogenic substances, such as Vascular Endothelial Growth Factor (VEGF) [7], Fibroblast Growth Factor-2 (FGF-2) and tryptase. Among them tryptase it is the most represented protein stored in MCs secretory granules and it can be secreted following c-Kit-R activation. In vitro studies indicated that tryptase it own strong angiogenic properties stimulating endothelial cells (ECs) to proliferate in both matrigel and chick embryo chorioallantoic membrane systems. In the last system, the addition of tryptase inhibitors suppressed ECs proliferation and then new blood microvessel formation. At cellular level tryptase binds protease-activated receptor-2 (PAR-2) that is a transmembrane G protein and then the signaling is internalized into the EC leading to their mitosis and finally new vessel formation [8–29].

With special regard to pacreatic cancer very little literature data have been available on this topic [30-32].

Here we aim to evaluate by mean of both immunohistochemial and morphometrical assay the density of mast cells positive to c-Kit receptor (c-Kit^+^ MCs), the density of MCs expressing tryptase (MCD-T) and microvessel density (MVD) in a series of 35 pancreatic ductal adenocarcinoma (PDAC) from patients underwent to up-front surgical treatment. In this research area, will be possible to evaluate our preliminary data for novel cancer biomarkers and for novel anti vascular approach in pancreatic tumor treatment.

## RESULTS

**Figure 1:**
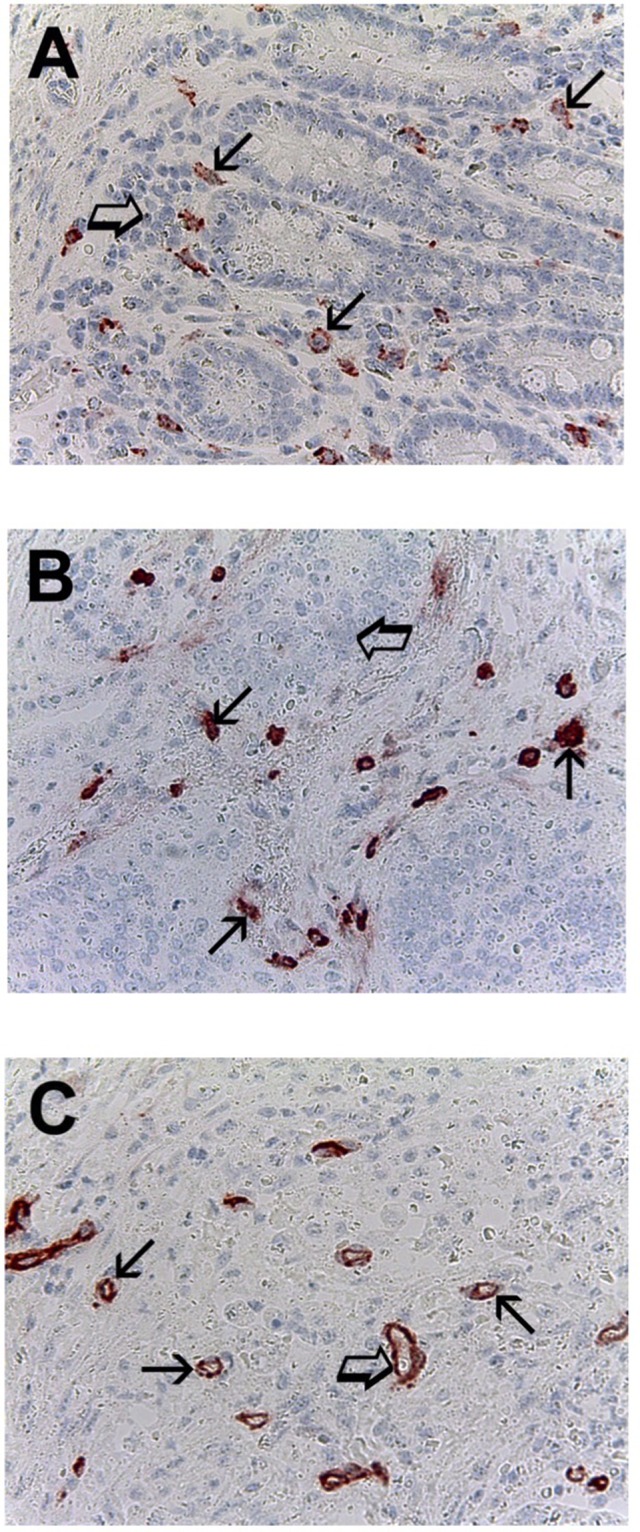
Pancreatic cancer tissue sections evaluated by immunohistochemistry **(A)** Many red immunostained mast cells positive to c-Kit receptor, arrows indicate single scattered mast cells, note the main membrane cytoplasmic immunostained pattern and the blue nucleus of mast cells is also observed. Big arrow indicates a clusters of pancreatic cancer cells (magnification x400). **(B)** Many red immunostained mast cells positive to tryptase, arrows indicate single scattered mast cells, big arrow indicates a cluster of pancreatic cancer cells (magnification x400). **(C)** Many red immunostained microvessels positive to anti-CD31 antibody, arrows indicate single scattered microvessels, big arrows indicate the red wall of microvessel with a hyaline translucent red blood cells in its lumen (magnification x400).

Immunohistochemistry assay utilizing the primary antibodies to c-Kit-R, to tryptase and to CD31 indicated that in more angiogenetic tumor area, so called *hot spots*, MCs are clearly identified close vessels (Figure 1).

The mean value ± standard deviation of c-Kit^+^ MCs, MCD-T and MVD was 13.23 ± 3.92, 12.47 ± 4.32 and 27.34 ± 8.97 respectively. In adjacent non tumoral and non inflamed pancreatic tissue the mean value of c-Kit^+^ MCs, MCD-T and MVD was 5.12 ± 2.10, 4.29 ± 1.20, 11.45 ± 8.59, respectively. These results demontstrated that c-Kit^+^ MCs, MCD-T and MVD are more represented in tumor tissue as compared to normal tissue.

**Figure 2:**
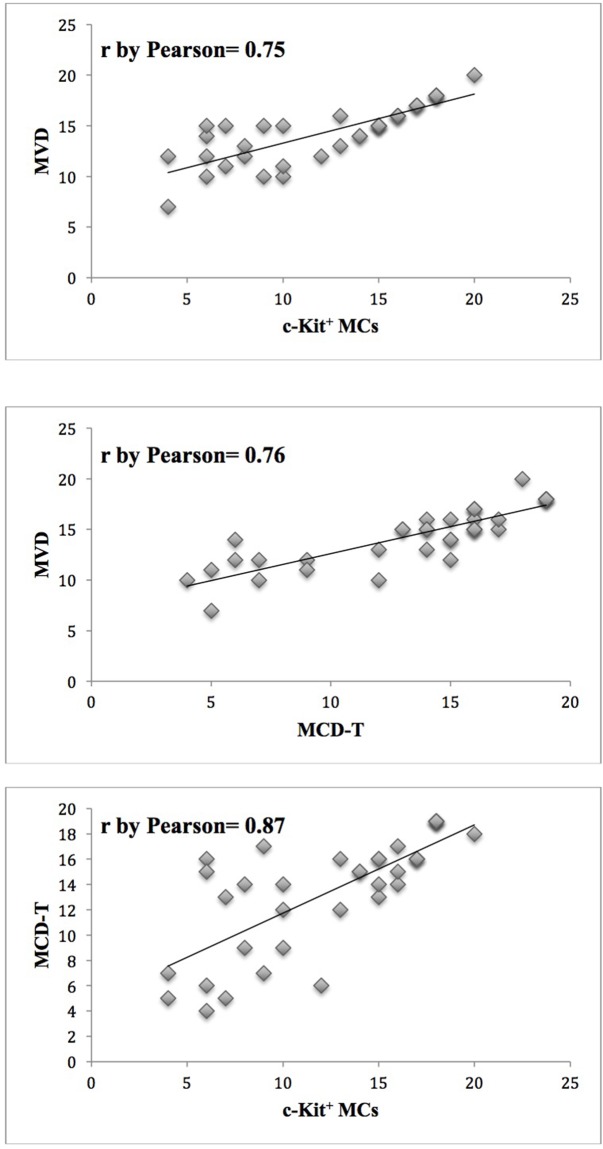
Correlation analysis between c-Kit^+^ MCs and MVD (r= 0.75, p= 0.04), MCD-T and MVD (r= 0.76, p= 0.03), between MCD-T and c-Kit^+^ MCs (r= 0.87 p= 0.01)

Significantly, in primary tumor tissue, the following association have been obtained between c-Kit^+^ MCs and MVD (r= 0.75, p= 0.04), MCD-T and MVD (r= 0.76, p= 0.03), MCD-T and c-Kit^+^ MCs (r= 0.87 p= 0.01) (Figure 2). No correlation concerning c-Kit^+^ MCs, MCD-T and microvessel density and the classical clinico-pathological hallmark has been discovered (data not shown). Cell count per field has been done at magnification 400x (0.19 mm^2^ area). Mean value difference between groups was measured by t test of Student and statistical significance was considered if p was ≤ 0.05.

## DISCUSSION

MCs are important in allergic and late-phase reactions, inflammation, and in the regulation of adaptive T-cell–mediated immunity. However, the role of MCs in cancers development is not totally understand, and data about their benefit or detriment to tumorigenesis have been controversial, depending on the local stromal conditions [33].

MCs could stimulate cancer development and progression in several ways. First, tumors can produce substances that attracting MCs in their microenvironment [33]. Second, in tumor microenvironment MCs activation can be triggered by the contact with tumor cells employing the pathway of adenosine/adenosine receptor [34]. Third MCs stimulation can be also triggered by cytokine and growth factor produced by tumor cells such as SCF that binding c-Kit-R lead to MCs activation and degranulation. [8, 35–36]. The final results is the exocytosis of pro-cancer and pro-angiogenetic mediators.

Among them tryptase it is the most represented protein stored in MCs secretory granules. In vitro studies indicated that tryptase it own strong angiogenic properties stimulating ECs to proliferate in both matrigel and chick embryo chorioallantoic membrane systems. In the last system, the addition of tryptase inhibitors suppressed ECs proliferation and then new blood microvessel formation. Tryptase is able to binds PAR-2 on endothelium stimulating the last to proliferate [8].

In other way tryptase indirectly stimulates neovascularization activating matrix metallopreteinases that in turn degrade the extracellular matrix leading to discharge of angiogenic factors in it contained [37–40].

Recently, pilot research, suggested that MCs are involved in neovascularization of pancreatic cancer and lymph node metastasis. In this manner, MCs presence in primary tumor tissue could influence growth tumor and overall survival of patients [41–45].

In our study, we have first assessed the status of c-Kit^+^ MCs, MCD-T and MVD in a series of 35 PDACP underwent to surgery. The results of our study need to be considered with some degree of caution due to the small sample of selected T_2-3_-N_0-1_-M_0_ analyzed patients. We chose to focus on the above subset of patients in that they were patients candidate to an up-front surgery treatment. Further awaited confirmatory studies could be more informative extending the analysis to patients with any TNM stages to evaluate possible differences through tumor progression.

To overcame possible methodological bias, the evaluation of the above parameters has been performed by mean of an image analysis system at x400 magnifications in a well defined microscopic area of 0,19 mm^2^ as previously published in other tumors type [46]. Next tissue evaluated parameters have been correlated to each other and results demonstrated a strong correlation between MCs, tryptase and microvascular bed.

On the other hand no correlation with the main clinico-pathological features has been found. From a biometrical point of view our results indicated that increased angiogenesis paralleled with both increased count of c-Kit^+^ MCs and MCD-T. It is interesting to underline that achieve data indicated a spatial localization of MCs mainly close vessels. Based on this histological location of MCs tryptase from them released could in loco act inducing microvessel formation and furthermore it could pass into blood flow facilitating tumor metastasis. In this scenario, tryptase could be degranulated from MCs following c-Kit-R activation. Based on these data we suggest that c-Kit^+^ MCs and MCD-T may be a novel surrogate angiogenic markers in pancreatic cancer patients.

From a translational and clinical point of view, it is intriguing to speculate the inhibition of pancreatic cancer angiogenesis at two different novel targets: first blocking MC activation employing available c-Kit-R inhibitors such as masitinib mesilate and second blocking tryptase utilizing gabexate mesilate or nafamostat mesilate [47–56]. Further studies in more large series of patients are awaited to confirm our preliminary data together with clinical trials aiming to evaluate the novel suggested therapeutic approaches regarding this very intriguingly topic.

## MATERIALS AND METHODS

### Study population

The clinico-pathological features of selected patients are summarized in Table 1. A total of 35 PDAC patients with stage T_2-3_N_0-1_M_0_ were undergone to potential curative resection. Surgical approaches used were: pancreaticoduodenectomy, distal pancreatectomy and total pancreatectomy with lymph node dissection. Patients were staged according to the American Joint Committee on Cancer 7^th^ edition (AJCC-TNM) classification and the World Health Organization classification (2000 version) was used for pathologic grading. All patients had not distant metastases on computed tomography. Full ethical approval and signed consent from individual patients was obtained. The study was conducted in accordance with the Declaration of Helsinki, and the protocol was approved by the Ethics Committee of the “Mater Domini” Hospital, “Magna Graecia” University, Catanzaro (N° 242; 22 December 2016).

**Table 1:** Clinico-pathological features of patients (*n*=35)

***Age***	
**► <65**	**25 (71%)**
**► >65**	**10 (29%)**
***Gender***	
**►Male**	**14 (40%)**
**►Female**	**21 (60%)**
***Tumour site***	
**►Head**	**15 (43%)**
**►Body-Tail**	**20 (57%)**
***TNM by AJCC stage***	
**►T_2_N_0-1_M_0_**	**16 (46%)**
**►T_3_N_0-1_M_0_**	**19 (54%)**
***Histologic type***	
**►Ductal adenocarcinomas**	**35 (100%)**
***Histologic grade***	
**►G1-G2**	**27 (77%)**
**►G3**	**8 (23%)**

### Immunohistochemistry

For the evaluation of c-Kit^+^ MCs, MCD-T and MVD a three-layer biotin-avidin-peroxidase system was utilized [57]. Briefly, 4 μm thick serial sections of formalin-fixed and paraffin-embedded surgical removed tumor samples were deparaffinised. Then, for antigen retrieval, sections were microwaved at 500W for 10 min, after which endogenous peroxidase activity was blocked with 3% hydrogen peroxide solution. Next, adjacent slides were incubated with the monoclonal antibodies anti-CD31 (QB-END 10; Bio-Optica Milan, Milan, Italy) for the identification of endothelial cells diluted 1:50 for 1 h at room temperature, antibodies anti-c-Kit-R (CD117; Dako) for 30 min and pH 8 for the identification of c-Kit^+^ MCs and antibodies anti-tryptase (clone AA1; Dako, Glostrup, Denmark) for the identification of MCD-T diluted 1:100 for 1 h at room temperature. The bound antibody was visualiszed using biotinylated secondary antibody, avidin-biotin peroxidase complex and fast red. Nuclear counterstaining was performed with Gill's haematoxylin no. 2 (Polysciences, Warrington, PA, USA). Primary antibody was omitted in negative controls.

### Morphometrical assay

Light microscopy integrated with an image analysis system (AXIO, Scope A1, ZEISS, Gottingen, Germany) was utilized.

Hot spot areas were selected at low magnification. C-Kit^+^ MCs, MCD-T and MVD were counted at x400 magnification (0.19 mm^2^ area, Figure 1A, 1B, 1C) [46]. In serial sections, each single c-Kit^+^ MCs and positive to tryptase was counted.

### Statistical analysis

Linear correlations between c-Kit^+^ MCs, MCD-T and MVD groups each to other were quantified by means of the Pearson’s correlation analysis. Difference between groups was measured by student t test and values were significantly different with p≤ 0.05.

Correlation among c-Kit^+^ MCs, MCD-T and MVD groups and the main clinico-pathological features were analysed by chi-square-test (χ2). All statistical analysis was performed with the SPSS statistical software package (SPSS, Inc., Chicago, IL).

## References

[1] Gilbert JA, Adhikari LJ, Lloyd RV, Halfdanarson TR, Muders MH, Ames MM (2013). Molecular markers for novel therapeutic strategies in pancreatic endocrine tumors. Pancreas.

[2] Daum O, Klecka J, Ferda J, Treska V, Vanecek T, Sima R, Mukensnabl P, Michal M (2005). Gastrointestinal stromal tumor of the pancreas: case report with documentation of KIT gene mutation. Virchows Arch.

[3] Patruno R, Marech I, Zizzo N, Ammendola M, Nardulli P, Gadaleta C, Introna M, Capriuolo G, Rubini RA, Ribatti D, Gadaleta CD, Ranieri G (2014). C-Kit expression, angiogenesis, and grading in canine mast cell tumour: a unique model to study c-Kit driven human malignancies. Biomed Res Int.

[4] Besmer P, Murphy JE, George PC, Qiu FH, Bergold PJ, Lederman L, Snyder HW, Brodeur D, Zuckerman EE, Hardy WD (1986). A new acute transforming feline retrovirus and relationship of its oncogene v-kit with the protein kinase gene family. Nature.

[5] Yarden Y, Kuang WJ, Yang-Feng T, Coussens L, Munemitsu S, Dull TJ, Chen E, Schlessinger J, Francke U, Ullrich A (1987). Human proto-oncogene c-kit: a new cell surface receptor tyrosine kinase for an unidentified ligand. EMBO J.

[6] Varricchi G, Galdiero MR, Marone G, Granata F, Borriello F, Marone G (2017). Controversial role of mast cells in skin cancers. Exp Dermatol.

[7] Detoraki A, Staiano RI, Granata F, Giannattasio G, Prevete N, de Paulis A, Ribatti D, Genovese A (2009). Vascular endothelial growth factors synthesized by human lung mast cells exert angiogenic effects. J Allergy Clin Immunol.

[8] Blair RJ, Meng H, Marchese MJ, Ren S, Schwartz LB, Tonnesen MG, Ribatti D, Genovese A, Triggiani M, Marone G (1997). Human mast cells stimulate vascular tube formation. Tryptase is a novel, potent angiogenic factor. J Clin Invest.

[9] Ammendola M, Sacco R, Sammarco G, Donato G, Montemurro S, Ruggieri E, Patruno R, Marech I, Cariello M, Vacca A, Gadaleta CD, Ranieri G (2014). Correlation between serum tryptase, mast cells positive to tryptase and microvascular density in colo-rectal cancer patients: possible biological-clinical significance. PLoS One.

[10] Ammendola M, Sacco R, Sammarco G, Donato G, Zuccalà V, Romano R, Luposella M, Patruno R, Vallicelli C, Verdecchia GM, Cavaliere D, Montemurro S, Ranieri G (2013). Mast cells positive to tryptase and c-kit receptor expressing cells correlates with angiogenesis in gastric cancer patients surgically treated. Gastroenterol Res Pract.

[11] Ammendola M, Sacco R, Sammarco G, Donato G, Zuccalà V, Luposella M, Patruno R, Marech I, Montemurro S, Zizzo N, Gadaleta CD, Ranieri G (2014). Mast cells density positive to tryptase correlates with angiogenesis in pancreatic ductal adenocarcinoma patients having undergone surgery. Gastroenterol Res Pract.

[12] Ammendola M, Sacco R, Marech I, Sammarco G, Zuccalà V, Luposella M, Patruno R, Giordano M, Ruggieri E, Zizzo N, Gadaleta CD, Ranieri G (2015). Microvascular density and endothelial area correlate with Ki-67 proliferative index in surgically-treated pancreatic ductal adenocarcinoma patients. Oncol Lett.

[13] Ammendola M, Marech I, Sammarco G, Zuccalà V, Luposella M, Zizzo N, Patruno R, Crovace A, Ruggieri E, Zito AF, Gadaleta CD, Sacco R, Ranieri G (2015). Infiltrating mast cells correlate with angiogenesis in bone metastases from gastric cancer patients. Int J Mol Sci.

[14] Ammendola M, Leporini C, Marech I, Gadaleta CD, Scognamillo G, Sacco R, Sammarco G, De Sarro G, Russo E, Ranieri G (2014). Targeting mast cells tryptase in tumor microenvironment: a potential antiangiogenetic strategy. Biomed Res Int.

[15] Ammendola M, Zuccalà V, Patruno R, Russo E, Luposella M, Amorosi A, Vescio G, Sammarco G, Montemurro S, De Sarro G, Sacco R, Ranieri G (2013). Tryptase-positive mast cells and angiogenesis in keloids: a new possible post-surgical target for prevention. Updat Surg.

[16] Ammendola M, Sacco R, Sammarco G, Piardi T, Zuccalà V, Patruno R, Zullo A, Zizzo N, Nardo B, Marech I, Crovace A, Gadaleta CD, Pessaux P (2016). Mast cells positive to tryptase, endothelial cells positive to protease-activated receptor-2, and microvascular density correlate among themselves in hepatocellular carcinoma patients who have undergone surgery. Onco Targets Ther.

[17] Ammendola M, Patruno R, Sacco R, Marech I, Sammarco G, Zuccalà V, Luposella M, Zizzo N, Gadaleta C, Porcelli M, Gadaleta CD, Ribatti D (2016). Ranieri G3. Mast cells positive to tryptase and tumour-associated macrophages correlate with angiogenesis in locally advanced colorectal cancer patients undergone to surgery. Expert Opin Ther Targets.

[18] Ammendola M, Sacco R, Vescio G, Zuccalà V, Luposella M, Patruno R, Zizzo N, Gadaleta C, Marech I, Ruggieri R, Kocak IF, Ozgurtas T, Gadaleta CD (2017). Tryptase mast cell density, protease- activated receptor-2 microvascular density, and classical microvascular density evaluation in gastric cancer patients undergoing surgery: possible translational relevance. Ther Adv Gastroenterol.

[19] Gulubova M, Vlaykova T (2009). Prognostic significance of mast cell number and microvascular density for the survival of patients with primary colorectal cancer. J Gastroenterol Hepatol.

[20] Marech I, Ammendola M, Sacco R, Capriuolo GS, Patruno R, Rubini R, Luposella M, Zuccalà V, Savino E, Gadaleta CD, Ribatti D1, Ranieri G (2014). Serum tryptase, mast cells positive to tryptase and microvascular density evaluation in early breast cancer patients: possible translational significance. BMC Cancer.

[21] Ranieri G, Ammendola M, Patruno R, Celano G, Zito FA, Montemurro S, Rella A, Di Lecce V, Gadaleta CD, Battista De Sarro G, Ribatti D (2009). Tryptase-positive mast cells correlate with angiogenesis in early breast cancer patients. Int J Oncol.

[22] Ranieri G, Ammendola M, Marech I, Laterza A, Abate I, Oakley C, Vacca A, Sacco R, Gadaleta CD (2015). Vascular endothelial growth factor and tryptase changes after chemoembolization in hepatocarcinoma patients. World J Gastroenterol.

[23] Ribatti D, Ranieri G, Nico B, Benagiano V, Crivellato E (2011). Tryptase and chymase are angiogenic *in vivo* in the chorioallantoic membrane assay. Int J Dev Biol.

[24] Stack MS, Johnson DA (1994). Human mast cell tryptase activates single-chain urinary-type plasminogen activator (pro-urokinase). J Biol Chem.

[25] Fajardo I, Pejler G (2003). Human mast cell beta-tryptase is a gelatinase. J Immunol.

[26] Itoh Y, Sendo T, Oishi R (2015). Physiology and pathophysiology of proteinase-activated receptors (P ARs): role of tryptase/P AR-2 in vascular endothelial barrier function. J Pharmacol Sci.

[27] Matej R, Mandàkovà P, Netikovà I, Poucková P, Olejár T (2007). Proteinase-activated receptor-2 expression in breast cancer and the role of trypsin on growth and metabolism of breast cancer cell line MDA MB-231. Physiol Res.

[28] Morris DR, Ding Y, Ricks TK, Gullapalli A, Wolfe BL, Trejo J (2006). Protease-activated receptor-2 is essential for factor VIIa and Xa-induced signaling, migration, and invasion of breast cancer cells. Cancer Res.

[29] Rickard A, Portell C, Kell PJ, Vinson SM, McHowat J (2005). Protease-activated receptor stimulation activates a Ca2+-independent phospholipase A2 in bladder microvascular endothelial cells. Am J Physiol Renal Physiol.

[30] Ma Y, Ullrich SE (2013). Intratumoral mast cells promote the growth of pancreatic cancer. Oncoimmunology.

[31] Soucek L, Lawlor ER, Soto D, Shchors K, Swigart LB, Evan GI (2007). Mast cells are required for angiogenesis and macroscopic expansion of Myc-induced pancreatic islet tumors. Nat Med.

[32] Strouch MJ, Cheon EC, Salabat MR, Krantz SB, Gounaris E, Melstrom LG, Dangi-Garimella S, Wang E, Munshi HG, Khazaie K, Bentrem DJ (2010). Crosstalk between mast cells and pancreatic cancer cells contributes to pancreatic tumor progression. Clin Cancer Res.

[33] Marone G, Varricchi G, Loffredo S, Granata F (2016). Mast cells and basophils in inflammatory and tumor angiogenesis and lymphangiogenesis. Eur J Pharmacol.

[34] Gorzalczany Y, Akiva E, Klein O, Merimsky O, Sagi-Eisenberg R (2017). Mast cells are directly activated by contact with cancer cells by a mechanism involving autocrine formation of adenosine and autocrine/paracrine signaling of the adenosine A3 receptor. Cancer Lett.

[35] Norrby K (2002). Mast cells and angiogenesis. APMIS.

[36] Wasiuk A, de Vries VC, Hartmann K, Roers A, Noelle RJ (2009). Mast cells as regulators of adaptive immunity to tumours. Clin Exp Immuno.

[37] Darmoul D, Marie JC, Devaud H, Gratio V, Laburthe M (2001). Initiation of human colon cancer cell proliferation by trypsin acting at protease-activated receptor-2. Br J Cancer.

[38] Donato G, Conforti F, Camastra C, Ammendola M, Donato A, Renzulli A (2014). The role of mast cell tryptases in cardiac myxoma: Histogenesis and development of a challenging tumor. Oncol Lett.

[39] Soreide K, Janssen EA, Körner H, Baak JP (2006). Trypsin in colorectal cancer: molecular biological mechanisms of proliferation, invasion, and metastasis. J Pathol.

[40] Liu Y, Mueller BM (2006). Protease-activated receptor-2 regulates vascular endothelial growth factor expression in MDA-MB-231 cells via MAPK pathways. Biochem. Biophys Res Commun.

[41] Cai SW, Yang SZ, Gao J, Pan K, Chen JY, Wang YL, Dong JH (2011). Prognostic significance of mast cell count following curative resection for pancreatic ductal adenocarcinoma. Surgery.

[42] Chang DZ, Ma Y, Ji B, Wang H, Deng D, Liu Y, Logsdon CD, Hwu P (2011). Mast cells in tumor microenvironment promotes the *in vivo* growth of pancreatic ductal adenocarcinoma. Clin Cancer Res.

[43] Esposito I, Menicagli M, Funel N, Bergmann F, Boggi U, Mosca F, Bevilacqua G, Campani D (2004). Inflammatory cells contribute to the generation of an angiogenic phenotype in pancreatic ductal adenocarcinoma. J Clin Pathol.

[44] Ammendola M, Sacco R, Zuccalà V, Luposella M, Patruno R, Gadaleta P, Zizzo N, Gadaleta CD, De Sarro G, Sammarco G, Oltean M, Ranieri G (2016). Mast cells density positive to tryptase correlate with microvascular density in both primary gastric cancer tissue and loco-regional lymph node metastases from patients that have undergone radical surgery. Int J Mol Sci.

[45] Ammendola M, Sacco R, Donato G, Zuccalà V, Russo E, Luposella M, Vescio G, Rizzuto A, Patruno R, De Sarro G, Montemurro S, Sammarco G, Ranieri G (2013). Mast cell positivity to tryptase correlates with metastatic lymph nodes in gastrointestinal cancer patients treated surgically. Oncology.

[46] Ranieri G, Labriola A, Achille G, Florio G, Zito AF, Grammatica L, Paradiso A (2002). Microvessel density, mast cell density and thymidine phosphorylase expression in oral squamous carcinoma. Int J Oncol.

[47] Marech I, Ammendola M, Gadaleta C, Zizzo N, Oakley C, Gadaleta CD, Ranieri G (2014). Possible biological and translational significance of mast cells density in colorectal cancer. World J Gastroenterol.

[48] Ammendola M, Sacco R, Sammarco G, Luposella M, Patruno R, Gadaleta CD, Sarro GD, Ranieri G (2016). Mast cell-targeted strategies in cancer therapy. Transfus Med Hemother.

[49] Deplanque G, Demarchi M, Hebbar M, Flynn P, Melichar B, Atkins J, Nowara E, Moyé L, Piquemal D, Ritter D, Dubreuil P, Mansfield CD, Acin Y (2015). A randomized, placebo-controlled phase III trial of masitinib plus gemcitabine in the treatment of advanced pancreatic cancer. Ann Oncol.

[50] Deplanque G, Demarchi M, Hebbar M, Flynn P, Melichar B, Atkins J, Nowara E, Moyé L, Piquemal D, Ritter D, Dubreuil P, Mansfield CD, Acin Y (2015). A randomized, placebo-controlled phase III trial of masitinib plus gemcitabine in the treatment of advanced pancreatic cancer. Ann Oncol.

[51] Erba F, Fiorucci L, Pascarella S, Menegatti E, Ascenzi P, Ascoli F (2001). Selective inhibition of human mast cell tryptase by gabexate mesylate, an antiproteinase drug. Biochem Pharmacol.

[52] Humbert M, Castéran N, Letard S, Hanssens K, Iovanna J, Finetti P, Bertucci F, Bader T, Mansfield CD, Moussy A, Hermine O, Dubreuil P (2010). Masitinib combined with standard gemcitabine chemotherapy: *in vitro* and *in vivo* studies in human pancreatic tumour cell lines and ectopic mouse model. PLoS One.

[53] Leporini C, Ammendola M, Marech I, Sammarco G, Sacco R, Gadaleta C, Oakley C, Russo E, De Sarro G, Ranieri G (2015). Targeting mast cells in gastric cancer with special reference to bone metastases. World J Gastroenterol.

[54] Marech I, Patruno R, Zizzo N, Gadaleta C, Introna M, Zito AF, Gadaleta CD, Ranieri G (2013). Masitinib (AB1010), from canine tumour model to human clinical development: Where we are?. Crit Rev Oncol Hematol.

[55] Marech I, Leporini C, Ammendola M, Porcelli M, Gadaleta CD, Russo E, De Sarro G, Ranieri G (2015). Classical and non-classical proangiogenic factors as a target of antiangiogenic therapy in tumor microenvironment. Cancer Lett.

[56] Mori S, Itoh Y, Shinohata R, Sendo T, Oishi R, Nishibori M (2003). Nafamostat mesilate is an extremely potent inhibitor of human tryptase. J Pharmacol Sci.

[57] Ranieri G, Grammatica L, Patruno R, Zito AF, Valerio P, Iacobellis S, Gadaleta C, Gasparini G, Ribatti D (2007). A possible role of thymidine phosphorylase expression and 5-fluorouracil increased sensitivity in oropharyngeal cancer patients. J Cell Mol Med.

